# New Instruments for the Engine by Means of Which Gold Fillings Can Be Rapidly and Securely Inserted

**Published:** 1881-09

**Authors:** 


### NEW INSTRUMENTS FOR THE ENGINE BY MEANS OF WHICH GOLD FILLINGS CAN BE RAPIDLY AND SECURELY INSERTED.

Dr. Herbst demonstrated his new method of filling teeth by filling a large cavity in the molar, which he held in his hand, in fourteen minutes with non-cohesive gold. The instruments, which were of a spindle, round, pear, and other shapes, were highly polished. They are to be used with the dental engine, and slowly revolved while packing soft foil, and when using cohesive gold, and only then, should they be revolved rapidly, as the rapid motion will make soft foil cohesive even when in the cavity.

The speaker took a piece of ivory in which was a gold filling that had been smoothly finished and immersed in water, and by the rapid rotation of the instruments, he made the surface cohesive and built on more gold. He afterwards inserted two proximal fillings with Wolrab's gold cylinders in the mouth of Dr. Budde of Darmstadt, in fifteen minutes. (*Deutsche Viertel-jahrschrift fur Zahnheilkunde for July* 1881.

